# ‘Splice-at-will’ Cas12a crRNA engineering enabled direct quantification of ultrashort RNAs

**DOI:** 10.1093/nar/gkaf002

**Published:** 2025-01-20

**Authors:** Xinrui Fei, Chao Lei, Wei Ren, Chenghui Liu

**Affiliations:** Key Laboratory of Applied Surface and Colloid Chemistry, Ministry of Education, Key Laboratory of Analytical Chemistry for Life Science of Shaanxi Province, School of Chemistry & Chemical Engineering, Shaanxi Normal University, 620 West Chang’an Avenue, Chang’an District, Xi’an, Shaanxi 710119, P.R. China; Key Laboratory of Applied Surface and Colloid Chemistry, Ministry of Education, Key Laboratory of Analytical Chemistry for Life Science of Shaanxi Province, School of Chemistry & Chemical Engineering, Shaanxi Normal University, 620 West Chang’an Avenue, Chang’an District, Xi’an, Shaanxi 710119, P.R. China; Key Laboratory of Applied Surface and Colloid Chemistry, Ministry of Education, Key Laboratory of Analytical Chemistry for Life Science of Shaanxi Province, School of Chemistry & Chemical Engineering, Shaanxi Normal University, 620 West Chang’an Avenue, Chang’an District, Xi’an, Shaanxi 710119, P.R. China; Key Laboratory of Applied Surface and Colloid Chemistry, Ministry of Education, Key Laboratory of Analytical Chemistry for Life Science of Shaanxi Province, School of Chemistry & Chemical Engineering, Shaanxi Normal University, 620 West Chang’an Avenue, Chang’an District, Xi’an, Shaanxi 710119, P.R. China

## Abstract

We present a robust ‘splice-at-will’ CRISPR RNA (crRNA) engineering mechanism that overcomes the limitations of clustered regularly interspaced short palindromic repeats (CRISPR)/Cas system in directly detecting ultrashort RNAs. In this strategy, an intact Cas12a crRNA can be split from almost any site of the spacer region to obtain a truncated crRNA (tcrRNA) that cannot activate Cas12a even after binding an auxiliary DNA activator. While splicing tcrRNAs with a moiety of ultrashort RNA, the formed combination can work together to activate Cas12a efficiently, enabling ‘splice-at-will’ crRNA engineering. Importantly, the ‘splice-at-will’ crRNA exhibits almost the same *trans*-cleavage activation efficiency as that of a conventional intact crRNA. Therefore, by rationally designing a DNA auxiliary activator with a conserved tcrRNA-complementary sequence and an arbitrary short RNA-of-interest recognition domain, a general sensing system is established that directly utilizes traditional DNA-activated Cas12a to detect ultrashort RNAs. This ‘splice-at-will’ crRNA engineering strategy could faithfully detect ultrashort RNA sequences as short as 6–8 nt, which cannot be achieved by conventional Cas12a and Cas13a systems. Additionally, through flexible splicing site design, our method can precisely distinguish single-base differences in microRNA and other short RNA sequences. This work has significantly expanded the Cas12a-based diagnostic toolbox and opened new avenues for ultrashort RNA detection.

## Graphical Abstract

**Graphical Abstract:**
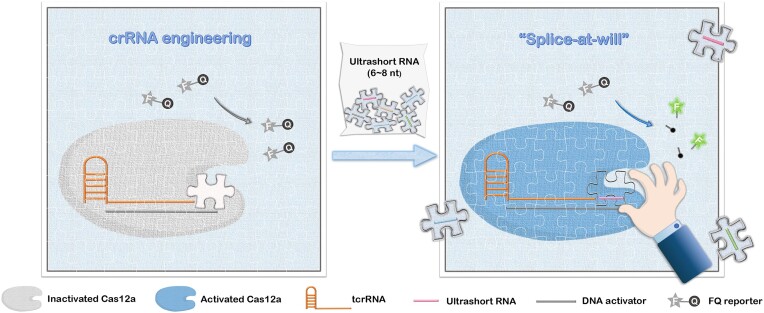

## Introduction

Small RNAs play important roles in gene expression (1), cell growth and development (2), and metabolism (3). The abnormal expression of some small RNAs, such as microRNAs (miRNAs) and ultrashort RNAs (<10 nt), is closely related to various diseases and biological activities (4–7). Notably, ultrashort RNAs, such as abortive transcripts (8) and pRNAs (9,10), are crucial participators and regulators in gene expression and various biological processes (11–13). Therefore, the detection of such small RNAs is of great significance for both biological studies and disease diagnostics. Conventionally, RNA quantification methods are generally realized by transcribing RNA targets into complementary DNA (cDNA) and further integrating various DNA amplification techniques (14–18). However, the quite short RNAs are generally poor substrates for yielding high-quality cDNAs via transcription (19–21). What is more, those shorter than 10 nt are even unable to be transcribed. Therefore, the accurate quantification of ultrashort RNAs is still challenging.

Due to the unique target-activated *trans*-cleavage activity, the clustered regularly interspaced short palindromic repeats (CRISPR)/CRISPR-associated (Cas) system, represented by Cas12a (22,23) and Cas13a (24,25), has been regarded as the next-generation tool for both DNA and RNA analyses (26–28). Nevertheless, as to the Cas12a sensing system, the *trans*-cleavage is generally DNA-activated, so RNA targets need to be pre-transcribed into DNA molecules (29–31). However, this reverse transcription process cannot work well on small RNA targets, especially the ultrashort ones. Though the Cas13a system can directly recognize RNA for activation, it requires a target RNA of at least ∼28 nt to efficiently activate its *trans*-cleavage nuclease activity (32,33), and an insufficient length of the target RNA will lead to a sharp decline in the detection performance (34). Therefore, up to now, neither conventional Cas12a-based nor Cas13a-based systems are feasible for the sensitive detection of ultrashort RNAs.

Herein, we presented a universal ‘splice-at-will’ CRISPR RNA (crRNA) engineering mechanism that enables Cas12a to directly detect RNA, particularly the ultrashort ones with high efficiency. In this strategy, with the assistance of a flexible DNA activator, ultrashort RNA can splice a sequence-conserved truncated crRNA (tcrRNA), forming a ‘splice-at-will’ crRNA with ∼100% Cas12a activation ability compared with conventional intact crRNA. Then, by rationally designing an auxiliary DNA activator with a conserved tcrRNA-complementary sequence and an arbitrary short RNA-of-interest recognition domain, a general sensing system has been established, enabling the conventional DNA-activated Cas12a to directly detect short RNAs. Small RNAs of various lengths, even the ultrashort ones under 10 nt, can be precisely quantified. Thus, this work addresses the challenges of detecting ultrashort RNAs that traditional Cas12a and Cas13a systems could not solve.

## Materials and methods

### Reagents and materials

EnGen LbaCas12a (Cpf1) and 10× NEBuffer 2.1 were ordered from New England Biolabs (MA, USA). 20× TE buffer (RNase-free, pH 7.5) and nuclease-free water were obtained from Thermo Fisher Scientific (WA, USA). 4S Red Plus Nucleic Acid Stain was supplied by Sangon Biotech (Shanghai, China). LwaCas13a was purchased from Kexin Biotech (Beijing, China). All DNA and RNA oligonucleotides were synthesized by either Thermo Fisher Scientific or Sangon Biotech and listed in Supplementary Table S1 and Supplementary Figures S1–S5.

### Standard procedures for investigating the ‘splice-at-will’ crRNA-induced Cas12a *trans*-cleavage activation and detecting miRNAs and ultrashort RNA

In general, the 10 μl whole reaction system includes the following two steps.

In the ‘splice-at-will’ crRNA assembly step, 25 nM tcrRNA, 25 nM DNA activators, let-7a (model target) with a series of concentrations and 2 mM MgCl_2_ were pre-hybridized in 1× NEBuffer 2.1 (10 mM Tris–HCl, 50 mM NaCl, 10 mM MgCl_2_, 100 μg/ml recombinant albumin, pH 7.9) at 90°C for 5 min, followed by slowly cooling to room temperature. The control sample was also generated by hybridizing an equivalent molar amount of tcrRNA and DNA activators without let-7a using the same annealing procedure. It should be noted that the concentration of model target let-7a was fixed at 5 nM when optimizing and investigating the influencing factors of ‘splice-at-will’ crRNA on Cas12a *trans*-cleavage activation. The procedure of detecting other RNAs is almost the same without further condition optimization, and it only needs to replace varying concentrations of let-7a with target RNAs (miRNA-17 and p8 6s-1) and corresponding DNA activators.

In the Cas12a cleavage reaction steps, the above pre-hybridization products, 1× NEBuffer 2.1, 25 nM LbaCas12a and 1 μM FQ reporter (5′-FAM-CCCCCCCCCC-BHQ1-3′) were mixed at 37°C for 50 min and then heated at 90°C for 3 min to inactivate the Cas12a. After adding 90 μl of 1× TE buffer (20 mM Tris–HCl, 1 mM EDTA, pH 7.9) to the reaction system, the fluorescence (ex. 488 nm, em. 500–600 nm) was measured using an F-7000 fluorescence spectrophotometer (Hitachi, Japan).

### Comparison of Cas12a relative activity induced by ‘splice-at-will’ crRNA and conventional full-length crRNA

For the ‘splice-at-will’ system, an equivalent molar amount of tcrRNA, DNA activators and let-7a fragment of only 9 nt (final concentration of 20 nM) were annealed in 1× NEBuffer 2.1 at 95°C for 5 min and then slowly cooled to 25°C. The sample of the conventional system was also generated by the hybridization of full-length intact crRNA and the normal DNA activator at the molar ratio of 1:1 using the same annealing procedure. Then, subsequent experiments for both samples proceeded according to the Cas12a cleavage reaction described above.

### Conventional Cas12a-based direct RNA detection

The reaction was conducted in a volume of 10 μl, including 1× NEBuffer 2.1, 25 nM crRNA, 25 nM LbaCas12a and 1 μM FQ reporter with varying concentrations of RNA target (let-7a or p8 6s-1) at 37°C for 50 min and then heated at 90°C for 3 min to inactivate the Cas12a. Then, 90 μl of 1× TE buffer was added to the reaction system and the fluorescence was measured using an F-7000 fluorescence spectrophotometer (Hitachi, Japan).

### Conventional Cas13a-based direct RNA detection

The reaction was conducted in a volume of 10 μl, including 1× reaction buffer (10 mM Tris–HCI, 1 mM DTT, 40 mM KCl, 3.5 mM MgCl_2_, pH 8.6), 25 nM crRNA, 25 nM LwaCas13a and 1 μM FQ reporter (5′-FAM-UUAUU-BHQ1-3′) with varying concentrations of RNA targets (let-7a or p8 6s-1) at 37°C for 50 min and then heated at 90°C for 5 min to inactivate Cas13a. Then, 90 μl of 1× TE buffer was added to the reaction system and the fluorescence was measured using an F-7000 fluorescence spectrophotometer (Hitachi, Japan).

## Results

### Feasibility and flexibility of Cas12a-tolerated ‘splice-at-will’ crRNA structure design

**Figure 1.**
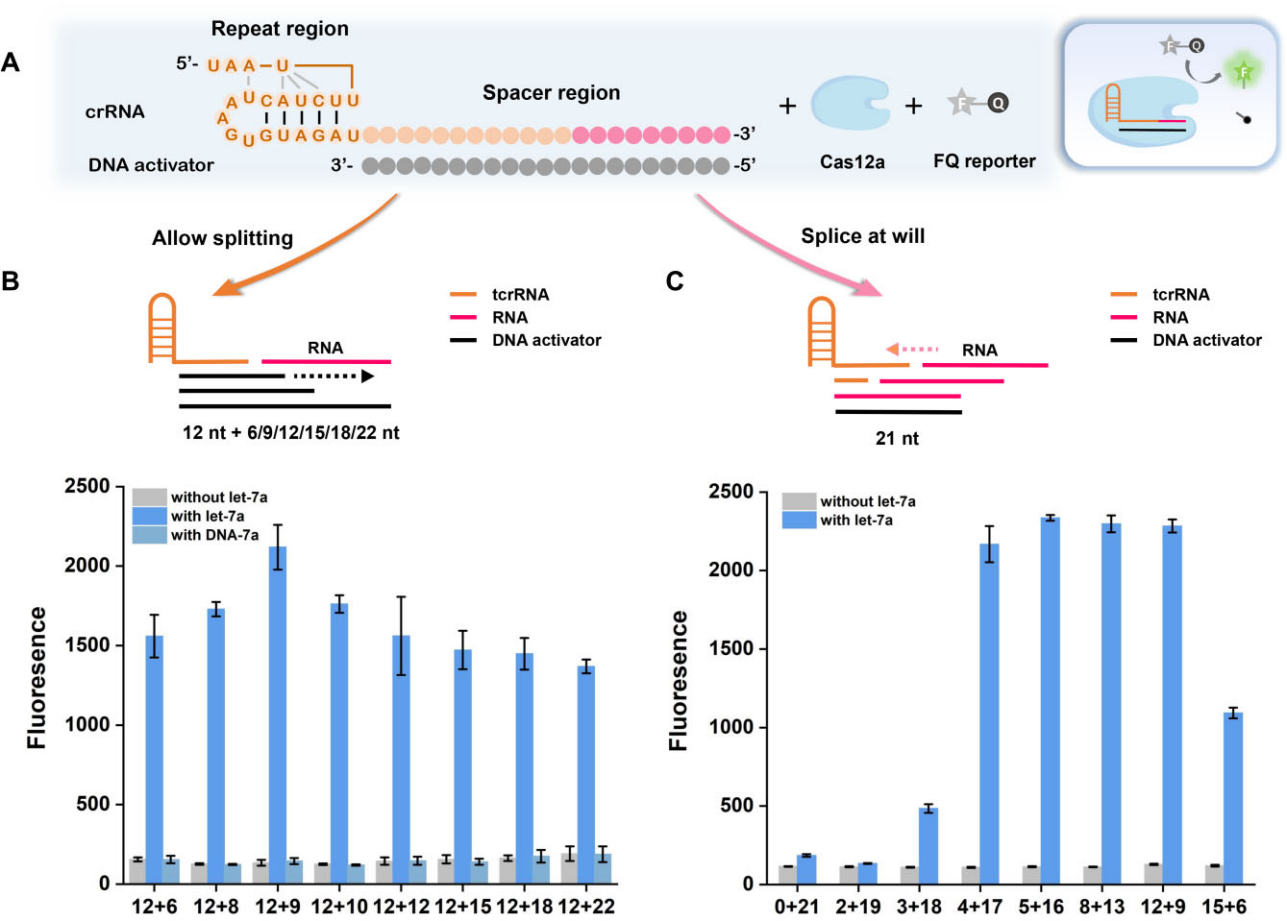
Investigating the effect of combination modes with different splicing lengths on ‘splice-at-will’ crRNA-induced Cas12a activation. (**A**) Schematic representation of a conventional crRNA activator complex inducing Cas12a *trans*-cleavage of FQ reporters. (**B**) Schematic representation of combining tcrRNA with RNA of different splicing lengths. The spacer length of tcrRNA is fixed at 12 nt and the splicing length of the RNA is designed from 6 to 22 nt. Corresponding fluorescence intensity of a series of 12+ *X* activators with different lengths (12 nt complementary to tcrRNA and *X* represents the number of nucleotides complementary to the RNA). (**C**) Schematic representation of splicing RNA with tcrRNAs of different spacer lengths. The DNA activator’s length was fixed at 21 nt. tcrRNA spacer length was designed from 0 to 15 nt, while the corresponding RNA splicing length was tuned from 21 to 6 nt. Corresponding fluorescence intensity induced by *X* + *Y* combination modes (*X* represents tcrRNA spacer length, while *Y* represents RNA splicing length). Error bars were calculated from triplicate experiments.

The formation of the Cas12a/crRNA/DNA activator ternary complex is the prerequisite to activate the *trans*-cleavage activity (35,36), which can efficiently cut the surrounding fluorophore/quencher double-labeled single-stranded DNA (ssDNA) reporter (FQ reporters) and generate strong fluorescence. A conventional Cas12a crRNA (37,38) comprises a handle-like repeat region with strict and conserved sequence, and a spacer region with its sequence complementary to the DNA activator (Figure 1A).

First, we investigated whether the splicing crRNA can effectively activate Cas12a (LbaCas12a) *trans*-cleavage activity. To test this, an intact crRNA is split from the middle of the spacer region, creating a tcrRNA motif that contains the Cas12a-binding repeat region and a short spacer region. Considering the hybridization stability, the tcrRNA spacer length was initially set to be 12 nt. As a conventional crRNA/DNA activator hybrid requires at least 17 nt to initiate the*trans*-cleavage (39), the tcrRNA cannot activate Cas12a after binding the DNA activator. On this basis, by adjusting the auxiliary DNA activator lengths, let-7a (40) was introduced as a proof-of-concept target with varying splicing lengths ranging from 6 nt at least to 22 nt of full length (Supplementary Figure S1A). As shown in Figure 1B, the tcrRNA alone was insufficient to activate Cas12a. When splicing let-7a with the tcrRNA, the formed combination with various lengths all worked well in Cas12a activation. Especially, the 12 + 9 combination generated the highest fluorescence signal. Upon substituting the RNA sequence of let-7a with the corresponding DNA sequence (DNA-7a), the *trans*-cleavage activity completely vanished irrespective of splicing length, indicating a strict RNA requirement for the splicing module. These results demonstrated that a truncated crRNA could cooperate with RNA targets of varying splicing lengths to rebuild the spacer region and form a full-function crRNA, in which the 21 nt spacer length works best in activating Cas12a *trans*-cleavage.

Then, we wonder whether crRNA can be truncated arbitrarily within the spacer region and flexibly splice an RNA target. For this, we designed tcrRNA with incomplete spacer lengths ranging from 0 to 15 nt and adjusted the corresponding let-7a splicing length from 21 to 6 nt under the optimal 21 nt spacer length, constructing a series of ‘splice-at-will’ crRNAs (Supplementary Figure S1B). As illustrated in Figure 1C, for the tcrRNAs that contain solely the repeat region, using let-7a as the entire spacer region failed to initiate LbaCas12a *trans*-cleavage, proving that splicing the two complete separated functional regions is not viable for LbaCas12a activation, while splicing let-7a with the tcrRNAs that own 3–15 nt spacers can initiate LbaCas12a *trans*-cleavage to varying degrees. Significantly, within a broad range of 4–12 nt of the tcrRNA spacers, the induced *trans*-cleavage was quite stable by showing high-intensity fluorescence signals. Overall, a short RNA target of interest can splice corresponding tcrRNAs truncated at almost any spacer site to form a ‘splice-at-will’ crRNA, thereby triggering Cas12a *trans*-cleavage with high efficiency.

Drawing from the above-mentioned explorations, the ‘splice-at-will’ crRNA structure design is universal and flexible, allowing Cas12a to be activated within a broad range of splicing modes. It should be noted that these explorations were all performed by precisely and adjacently splicing at the exact truncated site. Considering the diversity of RNA targets in the real detection, we further introduced overhang, three-way junction and gap at the splice joint site and tested their influence on the ‘splice-at-will’ crRNA system under the assistance of 12 + 9 DNA activator (Supplementary Figure S2A–D) that generates the highest fluorescence signal in the aforementioned test.

**Figure 2.**
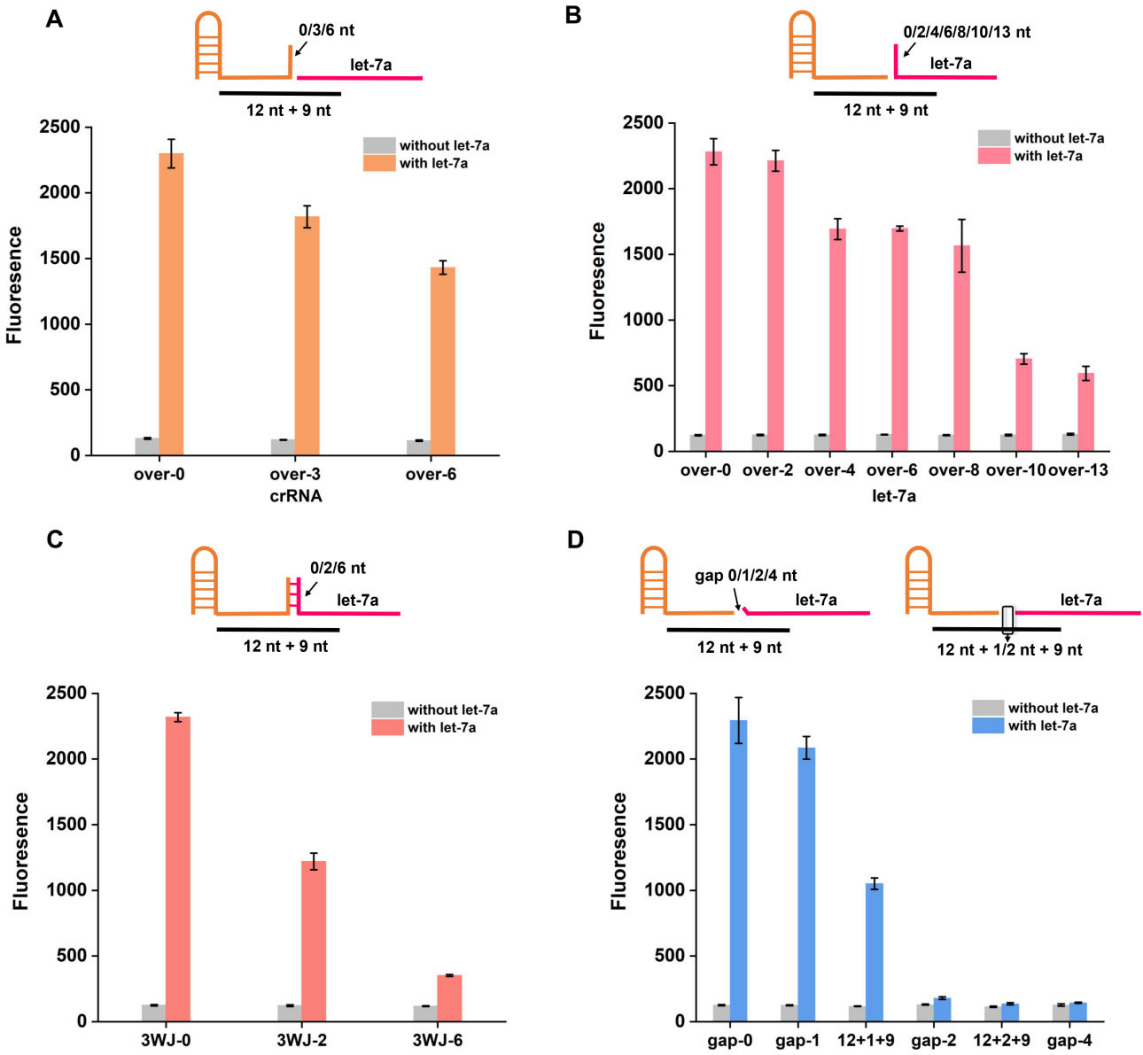
Investigating the effect of structural adjustments at the splice joint site on ‘splice-at-will’ crRNA-induced Cas12a *trans*-cleavage activity. Schematic illustration of splicing site containing different lengths of (**A**) tcrRNA overhang, (**B**) let-7a overhang, (**C**) three-way junction and (**D**) gap. *X* in gap-*X* represents the number of unmatched nucleotides between let-7a and 12 + 9 DNA activator, while *X* in 12 + X + 9 represents the number of extra added nucleotides in 12 + 9 DNA activators. Error bars were calculated from triplicate experiments.

As shown in Figure 2A and B, when long overhangs were produced by either extending tcrRNA or moving let-7a, the ‘splice-at-will’ crRNA maintained the capability of activating Cas12a *trans*-cleavage efficiently, albeit with slightly decreased fluorescence. Notably, the fluorescence signal was still apparent even when the let-7a overhang length reached its limit of 13 nt. These results show that the ‘splice-at-will’ crRNA is highly compatible with overhangs, which allows for various splicing modes of RNA targets. By further linking two overhangs (one from the tcrRNAs and the other from let-7a) to create a three-way junction (3WJ), we observed that the ‘splice-at-will’ crRNA containing 2–4 base pairs in the 3WJ structure retained the Cas12a activation capability despite the fluorescence signal decreasing obviously (Figure 2C). This suggested that the ‘splice-at-will’ crRNA can also tolerate the presence of a short 3WJ structure.

Then, we investigated the influence of splicing gap by constructing it in two ways: one is reducing the matched nucleotides at exactly the splicing site between let-7a and the 12 + 9 DNA activator to construct gap-*X* (*X* represents the number of unmatched nucleotides) and the other one is adding extra nucleotides in the 12 + 9 DNA activator while maintaining the matched length to construct 12 + *X* + 9 (*X* representing the number of extra added nucleotides). As shown in Figure 2D, no matter for gap-*X* or 12 + *X* + 9, when *X* is 2–4, ‘splice-at-will’ crRNA activation capability is almost completely suppressed. However, gap-1 and 12 + 1 + 9, with only 1 nt gap, could still cause strong fluorescence, implying that the ‘splice-at-will’ crRNA can tolerate a tiny gap. These distinct phenomena may suggest a promising way to precisely quantify single-base variations in short RNAs.

To sum up, ‘splice-at-will’ crRNA is compatible with flexible structure adjustments and broad splicing modes. For short RNA targets of varying lengths and sequences, splicing methods can be designed in an extremely flexible way to achieve efficient Cas12a activation and quantitative target detection, which provides a novel technique for ultrashort RNA detection.

### Mechanism of the ‘splice-at-will’ crRNA in activating Cas12a

**Figure 3.**
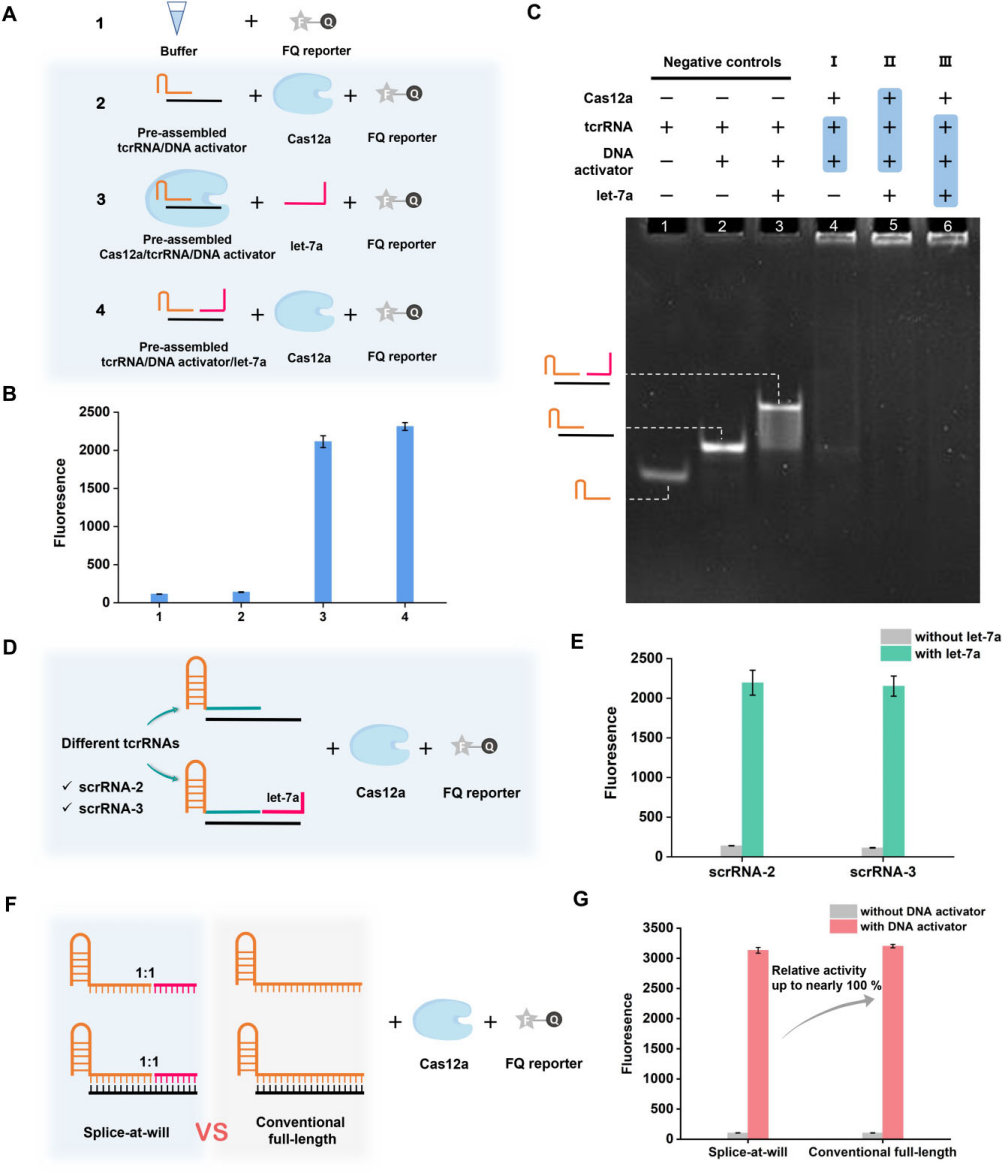
Investigating the activation mechanism and efficiency of the ‘splice-at-will’ crRNA on Cas12a *trans*-cleavage activity. (**A**) The schematic diagram of the reaction routes by pre-mixing 25 nM tcrRNA, 25 nM DNA activator, 25 nM Cas12a, 5 nM let-7a and FQ reporters in different orders. (**B**) Fluorescence responses generated by the above reaction routes. (**C**) Corresponding nondenaturing polyacrylamide gel electrophoresis (PAGE) results. The pre-assembled portions are highlighted. (**D**) The schematic diagram of investigating the universality of the ‘splice-at-will’ crRNA activation effects. (**E**) Fluorescence responses generated by ‘splice-at-will’ crRNA by use of the other two tcrRNAs (tcrRNA-2 and tcrRNA-3). (**F**) Schematic diagram of comparison of Cas12a relative activity induced by ‘splice-at-will’ crRNA and conventional full-length intact crRNA. tcrRNA, let-7a and full-length crRNA are all 20 nM. (**G**) Fluorescence responses generated by ‘splice-at-will’ crRNA and conventional full-length intact crRNA. Error bars were calculated from triplicate experiments.

The mechanism of the ‘splice-at-will’ crRNA in activating Cas12a *trans*-cleavage activity and its efficiency were further investigated. First, the interaction between the ‘splice-at-will’ crRNA and Cas12a was checked by pre-mixing tcrRNA, RNA target (let-7a), DNA activator and Cas12a in different orders (Figure 3A). As shown in Figure 3B, undigested FQ reporters produced a low fluorescence background in the buffer (panel 1). The same background appeared when the pre-assembled tcrRNA/DNA activator was mixed with Cas12a (panel 2), indicating the unactivated Cas12a *trans*-cleavage. In contrast, further adding let-7a regardless of the assembly orders, a strong fluorescence signal appeared (panel 3 and panel 4), suggesting that the RNA target (let-7a) plays a key role in the ‘splice-at-will’ crRNA/Cas12a interaction and *trans*-cleavage activation. The scenario of simultaneously mixing Cas12a, tcrRNAs, let-7a and DNA activator without any prior assembly was also investigated. The result of the simultaneous mixing is similar to the pre-assembled approach, albeit with a slightly lower activation efficiency of 94% compared to that in panel 4 (Supplementary Figure S6). This result suggests that the pre-assembly mode of panel 4 in the ‘splice-at-will’ system can most effectively activate the *trans*-cleavage efficiency of Cas12a. Meanwhile, the panel 3 mode also achieves a similar activation effect, indicating that when Cas12a pre-assembles with the tcrRNA/DNA activator, it can specifically search the matching RNA fragments. This process is similar to conventional CRISPR–Cas12a systems, where Cas12a along with the crRNA can specifically recognize and bind with the target DNA sequence through base pairing.

To understand the underlying principle, nondenaturing PAGE analysis was carried out. As displayed in Figure 3C, the tcrRNA/DNA activator heteroduplex could be clearly identified in lane 2. The addition of let-7a made the band upward, suggesting the formation of ‘splice-at-will’ crRNA (lane 3). When tcrRNA/DNA activator mixed with Cas12a, the band corresponding to that of tcrRNA/DNA activator heteroduplex almost disappeared (lane 4), indicating that the tcrRNA/DNA activator complex can inadequately bind with Cas12a in the absence of let-7a but yield a Cas12a-unactivated conformation. Compared with lane 4, further adding let-7a resulted in the disappearance of the residual tcrRNA/DNA activator bands and the distinct topmost bands, regardless of the assembly orders (lane 5 and lane 6), revealing that the RNA target promotes the full assembly of ‘splice-at-will’ crRNA and Cas12a. The above results from Figure 3A–C can be summarized as follows: regardless of assembly orders, in the absence of the RNA target, tcrRNA and DNA activator cannot fully bind with Cas12a and yield a Cas12a-unactivated state. However the addition of the RNA target to form ‘splice-at-will’ crRNA can efficiently bind with Cas12a. In this way, ‘splice-at-will’ crRNA can switch Cas12a to the activated state of the *trans*-cleavage activity.

Then, to verify whether this ‘splice-at-will’ crRNA activation mechanism is universal and sequence independent, two other random tcrRNA sequences (tcrRNA-2 and tcrRNA-3) were synthesized and tested (Figure 3D and Supplementary Figure S3A). As shown in Figure 3E, arbitrarily changing the tcrRNA spacer sequence in the ‘splice-at-will’ crRNA barely affected their Cas12a activation capability by showing high fluorescence intensities, suggesting the sequence independence of the ‘splice-at-will’ crRNA to induce stable Cas12a activation effect. On this basis, changing the corresponding spacer and auxiliary activator sequence according to the RNA sequence of interest can effectively activate Cas12a, which lays a foundation for the general detection of different RNA targets.

Finally, we compared the Cas12a activation efficiency by the ‘splice-at-will’ crRNA with that induced by a conventional full-length intact crRNA (Figure 3F). For this, we fine-tuned the ‘splice-at-will’ crRNA by integrating the tcrRNA with a truncated let-7a sequence of only 9 nt. Accordingly, a conventional full-length crRNA was designed with an identical spacer sequence to the ‘splice-at-will’ crRNA (Supplementary Figure S3B). As shown in Figure 3G, after adding the same ssDNA activator, the ‘splice-at-will’ crRNA produced almost the same fluorescence intensity as the conventional full-length intact crRNA, inducing up to 97.8% of relative *trans*-cleavage activity. This result indicated that taking advantage of the powerful ‘splice-at-will’ crRNA engineering, Cas12a *trans*-cleavage can be fully activated by ultrashort RNA sequences, the activity of which is as effective as that induced by traditional conventional intact crRNA/DNA activator.

### Sensitive detection of miRNA and ultrashort RNA by the ‘splice-at-will’ sensing system

**Figure 4.**
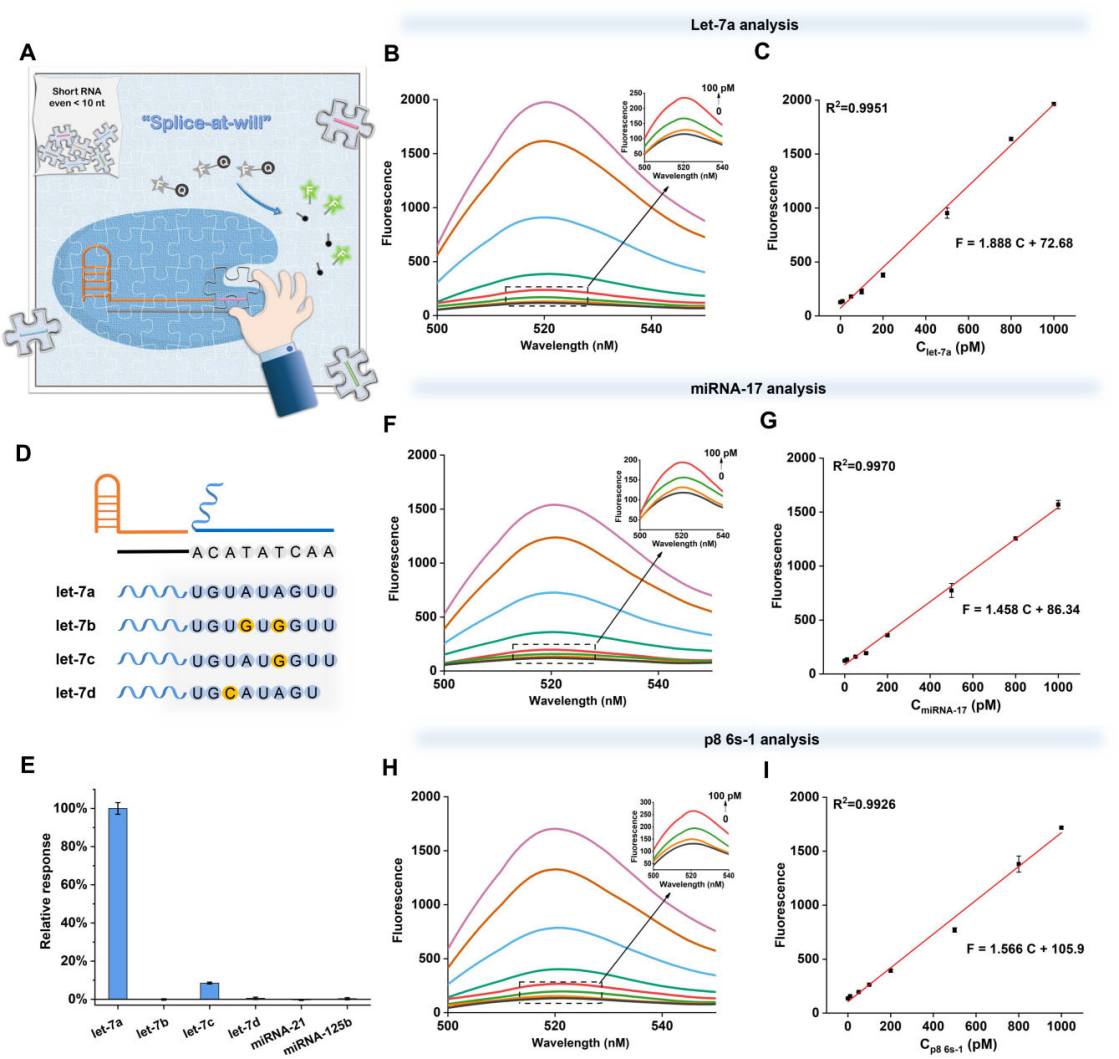
Direct detection of miRNA and ultrashort RNA by the ‘splice-at-will’ system. (**A**) Working principle of the sensing designs for p8 6s-1, let-7a and miRNA-17 analysis. (**B**) Fluorescence spectra of the system in response to different concentrations of let-7a. The concentrations of RNA targets are 10–1000 pM. (**C**) The linear relationship between fluorescence intensity and let-7a. (**D**) Schematic diagram depicting let-7 family miRNA mutations in the splicing region. (**E**) Relative fluorescence responses induced by let-7a and other miRNAs (5 nM). Error bars were calculated from triplicate experiments. (**F**) The fluorescence spectra of the system in response to different concentrations of miRNA-17. (**G**) The linear relationship in the miRNA-17 analysis. (**H**) The fluorescence spectra of the system in response to different concentrations of p8 6s-1. (**I**) The linear relationship in the p8 6s-1 analysis.

Based on the above-mentioned studies, a new and robust Cas12a ‘splice-activation’ strategy has been presented by constructing a ‘splice-at-will’ crRNA. This distinct ‘splice-at-will’ crRNA design ingeniously integrates a truncated crRNA motif and a short RNA sequence to activate Cas12a *trans*-cleavage. What is more, any RNA target can be flexibly inserted in the ‘splice-at-will’ crRNA by simply changing the DNA activator sequences that hybridize with the RNA target. Therefore, a ‘splice-at-will’ sensing system was further developed for the direct detection of various short RNA targets (Figure 4A).

First, the analytical performance of the system was tested by applying it to let-7a miRNA detection (Supplementary Figure S4A). According to Figure 4B, the fluorescence response of the system increased gradually with let-7a concentration increasing from 10 pM to 1 nM. A good linear relationship (with *R*^2^ = 0.9951) was obtained between the fluorescence intensity (at 518 nm) and let-7a concentration within this range (Figure 4C). With the limit of detection (LOD) of 4.8 pM, this system realized the sensitive detection of miRNA let-7a.

Next, the selectivity of the system was evaluated by checking its capacity to distinguish single-base differences. It should be noted that the conventional Cas12a system has a poor ability to distinguish single-base mutations in non PAM regions (41). However, according to our previous results in Figure 2, the gap in the ‘splice-at-will’ crRNA can greatly affect its performance on activating Cas12a, so our method is very sensitive to the single-base mismatches, allowing for accurately distinguishing those within the recognizing region. To verify it, we tested and compared let-7a with other miRNAs, including homologous let-7 family members (let-7b with two-base difference, let-7c with one-base difference, let-7d with one-base difference) and nonhomologous ones (miRNAs miRNA-21 and miR-125b). Taking advantage of ‘splice-at-will’ crRNA tolerance to various splicing modes of RNA targets, let-7a family mutations can be put in the splicing region by designing the corresponding DNA activator sequence (Figure 4D and Supplementary Figure S4B). As shown in Figure 4E, a strong fluorescence only appeared with the participation of let-7a. A series of interfering miRNA molecules, including even one- or two-base mismatched let-7 family miRNAs, were well distinguished (interference <9%). Notably, for let-7d with onw-base difference and let-7b with two-base difference in the splicing region, their interferences were negligible, which indicates the high specificity of the ‘splice-at-will’ sensing system.

Finally, to demonstrate the versatility of the detection system, we also tested miRNA-17, a cancer biomarker (42), and obtained a similar result (Figure 4F and G) with an LOD of 6.0 pM. Considering that the above explorations all used the 9 nt moieties in miRNAs as the binding parts of splicing crRNA, to verify that our methodology can be really and truly used for ultrashort RNA sequence detection, the ‘splice-at-will’ system was further applied to detect p8 6s-1 RNA of only 8 nt length without systematically optimizing assay condition (Supplementary Figure S4C). As a product RNA of the global bacterial transcription regulator, p8 6s-1 plays an important role in investigating bacterial growth and modulating stress as well as optimizing survival during nutrient limitation (9,10,13). As shown in Figure 4H and I, p8 6s-1 with only 8 nt length could be easily detected with an LOD of 4.8 pM. In conclusion, the ‘splice-at-will’ sensing system provides a simple, flexible, sensitive, specific and robust approach for the detection of various small RNA targets, even ultrashort RNAs.

### The distinct advantage of the ‘splice-at-will’ sensing system for ultrashort RNA detection

**Figure 5.**
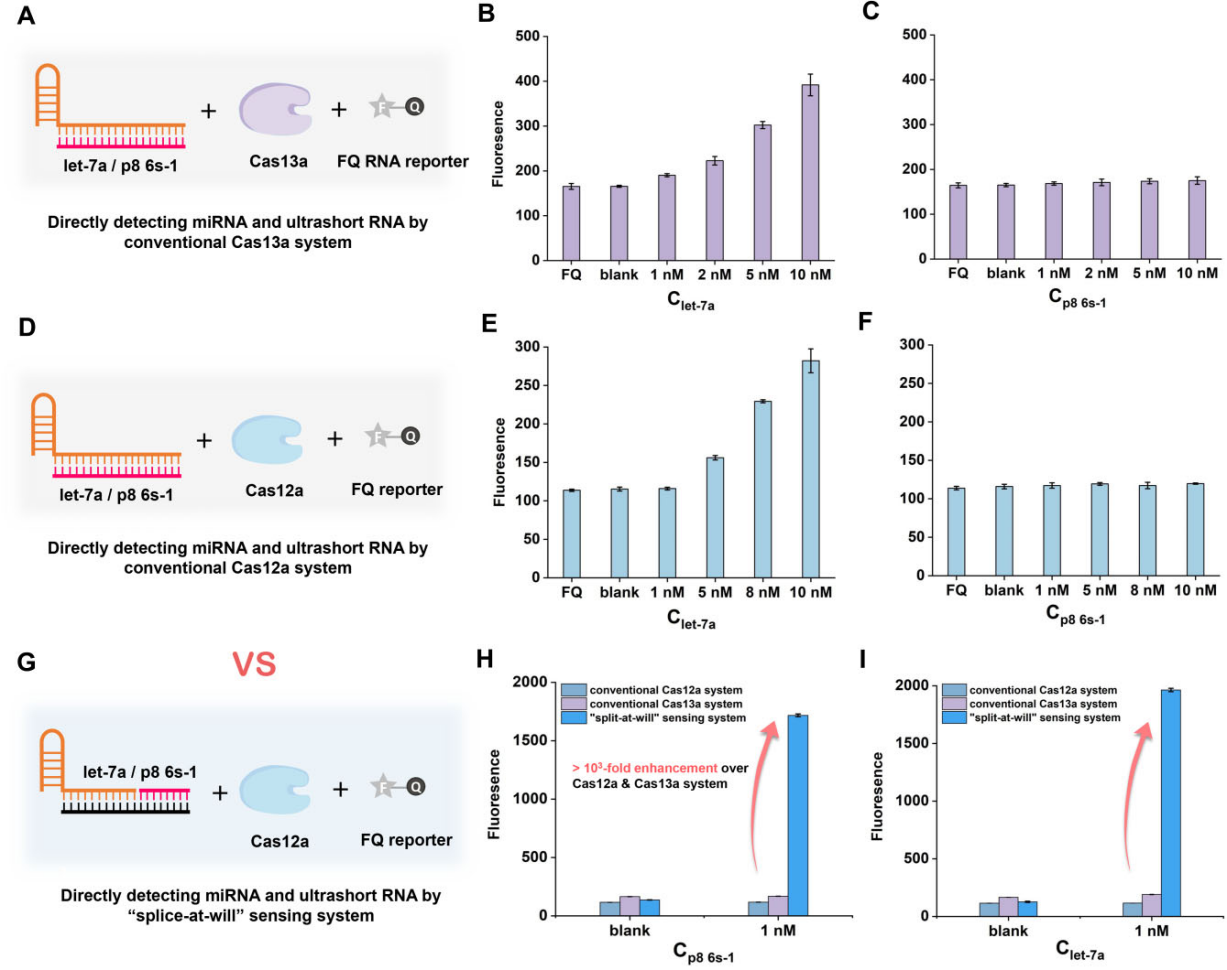
Comparison of the performance of the ‘splice-at-will’ sensing system with conventional Cas13a and Cas12a systems. (**A**) Schematic diagram of directly detecting miRNA and ultrashort RNA by a conventional Cas13a system. Corresponding fluorescence response of the Cas13a system to let-7a (**B**) and p8 6s-1 (**C**). (**D**) Schematic diagram of directly detecting miRNA and ultrashort RNA by a conventional Cas12a system. Corresponding fluorescence response of Cas12a system to let-7a (**E**) and p8 6s-1 (**F**). (**G**) Schematic diagram of directly detecting miRNA and ultrashort RNA by the ‘splice-at-will’ sensing system. Comparison of fluorescence responses of the ‘splice-at-will’ sensing system and conventional Cas12a/Cas13a systems in detecting 1 nM p8 6s-1 (**H**) and 1 nM let-7a (**I**). Error bars were calculated from triplicate experiments.

To emphasize the superior performance of the ‘splice-at-will’ sensing system, we compared it with other conventional CRISPR/Cas systems by detecting miRNA let-7a and ultrashort RNA p8 6s-1. In conventional *trans*-cleavage assay, let-7a and p8 6s-1 served as activators to directly bind crRNA of equal length. First, a conventional Cas13a system was tested, which is widely considered suitable for RNA detection due to its RNA-activated *trans*-cleavage property (Figure 5A and Supplementary Figure S5A). However, the standard LwaCas13a system requires a target RNA of 28 nt for full activation (32,33). For let-7a with 22 nt, the apparent signals could be observed only when the concentration of let-7a reached at least the nanomolar level under our normal experimental conditions (Figure 5B). More critically, 8 nt p8 6s-1 of different concentrations were all undetectable, receiving no response from the Cas13a system (Figure 5C). Then, the conventional Cas12a system was tested (Figure 5D and Supplementary Figure S5B), which is traditionally deemed to be DNA-activated, but recently shown to be RNA-activated as well (43–45), although the efficiency is still disputed. Under the same common condition, let-7a of over 5 nM could be distinguished from the blank control with poor RNA-activated efficiency than Cas13a (Figure 5E), which is consistent with the previous study (45). Meanwhile, Cas12a still failed to detect p8 6s-1 of different concentrations (Figure 5F). In contrast, by utilizing our ‘splice-at-will’ sensing system, p8 6s-1 as low as 10 pM can be clearly detected. In particular, it can be seen from Figure 5G–I that the fluorescence response produced by 1 nM p8 6s-1 is much stronger (>10^3^-fold) than that produced by fluorescence response than conventional Cas12a and Cas13a systems, and the detection of let-7a obtained a similar result.

The practicable detection performance of our system was further tested in total small RNA samples extracted from HeLa cells. The amount of let-7a in 100 ng of total small RNA samples was calculated to be 300 pM (in 10 μl), consistent with the results of the quantitative reverse transcription polymerase chain reaction assay (330 pM, Supplementary Figure S7). Since the amount of p8 6s-1 in total small RNA samples is negligible, the recovery test was subsequently conducted by spiking 100 pM of synthetic p8 6s-1 into 20 ng total small RNA samples. A good recovery result (96.3%) was obtained as shown in Supplementary Table S2, indicating the reliable performance of our system for short RNA analysis in real complex samples. To sum up, the ‘splice-at-will’ sensing system shows a distinct advantage in accurately quantifying ultrashort RNAs, which cannot be achieved by traditional Cas12a and Cas13a systems. As a new powerful tool for the Cas sensing system, our splicing strategy effectively solves the challenging problem of ultrashort RNA detection in a simple and convenient way, broadening the toolbox of traditional Cas-based diagnostic technologies.

## Discussion

CRISPR/Cas technology, especially Cas12a and Cas13a, has become the powerful next-generation tool for nucleic acid-based molecular diagnostics. Nevertheless, they are not feasible for the sensitive detection of ultrashort RNAs because the widely used Cas12a is typically activated by DNA and Cas13a requires longer RNA for activation.

Currently, several pioneering studies have attempted to expand the application scenarios of Cas systems by rational activator/crRNA engineering. For example, Jain, Liu and coworkers (43–47) reported that RNA targets can be tolerated as a part of the activator in the Cas12a system when accompanied by a short ssDNA or a PAM-containing double-stranded DNA and even as the whole activator alone. However, the Cas12a *trans*-cleavage activity can just be partially initiated, much less effectively than that induced by traditional DNA activators (43,48). On the other hand, a preliminary study has reported the crRNA engineering strategy by completely and exactly splitting the Cas12a-binding repeat region (termed ‘scaffold RNA’) and the whole target-recognizing spacer region (termed ‘spacer RNA’), which extends the Cas12a function for RNA target sensing (49). Nevertheless, it requires extremely high concentrations (hundreds of nanomolar) of Cas12a and scaffold RNA to activate the *trans*-cleavage activity. Also, this strategy may be only applied to given kinds of Cas12a (e.g. AsCas12a) and does not work well with widely used LbaCas12a (50). Moreover, the assembly of Cas12a and completely separated scaffold RNA loses the ability to further specifically recruit spacer RNA and cannot achieve the sensitive detection of ultrashort RNAs. Therefore, although such Cas engineering mechanisms are quite promising, the CRISPR/Cas-based detection of ultrashort RNAs remains unsolved.

In this work, we have developed a new and robust ‘splice-at-will’ crRNA engineering mechanism that enables Cas12a to directly detect ultrashort RNAs. We discovered that an intact Cas12a crRNA can be truncated at almost any spacer site to yield a tcrRNA that cannot activate Cas12a activity after binding a DNA activator. While splicing the tcrRNA with a short RNA, the formed ‘splice-at-will’ crRNA can efficiently activate Cas12a *trans*-cleavage ability. Even for the tcrRNA sequence with only 4 nt spacer length or only 6 nt length of ultrashort RNAs, the formed ‘splice-at-will’ crRNA can stably activate Cas12a activity due to auxiliary DNA-mediated hybridization and Cas12a’s inherently efficient recognition and stabilizing effect on the splicing crRNA. Notably, we also checked the possibility of splicing RNA targets with tcrRNAs consisting solely of the repeat region. We found that LbaCas12a *trans*-cleavage activity was not activated at all, suggesting that the above-mentioned complete separation of scaffold RNA and spacer RNA-based crRNA engineering is inapplicable to LbaCas12a under the normal Cas12a *trans*-cleavage-based sensing conditions. This also underscores the importance of retaining certain key sequences and structural features within the tcrRNA spacer region, particularly the seed region, to ensure the effective binding and activation of the Cas12a protein. Thus, the ‘splice-at-will’ crRNA engineering mechanism is totally a new breakthrough that well addresses the big challenges of ultrashort RNA sensing.

Compared with conventional Cas12a-based sensing systems, this ‘splice-at-will’ crRNA engineering mechanism generates almost identical Cas12a activation efficiency under common conditions without any special requirements. By simply transforming the binary assembly form of crRNA/DNA activator into a new type of ternary assembly form, it enables the traditionally DNA-activated Cas12a to directly detect miRNA and even ultrashort RNA of only 6–8 nt with high sensitivity, which cannot be achieved by existing Cas12a- and even Cas13a-based methods.

Additionally, this ‘splice-at-will’ splicing model allows for arbitrary and flexible assembly of RNA modules and corresponding DNA activators, where users have full control over the sequence and length of the splicing model, making it universally applicable to the analysis of various RNA sequences. Theoretically, any RNA sequence can be detected generally as long as the auxiliary DNA activator sequence follows the elucidated design rules to meet a rational length comprising a tcrRNA-complementary region and an RNA-targeting region. At the same time, the formed combination is extremely sensitive to the various designs, including the setting of splicing sites, single-base differences in the splicing region and even the inserted gaps at the splice joint site. Through flexible splicing design, even the single nucleotide polymorphisms in the let-7a families can be well distinguished. Thus, this method also provides a more general and flexible solution for the precise single nucleotide discrimination of short RNA targets.

To better describe the ‘splice-at-will’ design, the optimal splicing principles are summarized in detail. First, for the length of the splicing mode (*X* + *Y*), neither the tcrRNA spacer region (*X*) nor the target RNA splicing region (*Y*) should be too short. Optimally, *X* should be selected within the range of 4–12 nt, and *Y* within 6–22 nt. The overall length of ‘*X* + *Y*’ is most effective when it falls within the range of 18–24 nt. Second, regarding the structure of the splicing mode, it should avoid the presence of gaps and overhangs near the splicing joint site to minimize the impact of structural variants on the splicing process. Third, since the optimal splicing modes may slightly vary according to the target sequence, one can flexibly select the most suitable design to achieve the best detection performance.

Finally, it is worth noting that in this work, we mainly concentrate on the construction of the short RNA splicing-activation mode and the in-depth exploration of its influencing factors. So, the detection performance is not yet fully explored. However, considering that the ultrashort RNA detection performance of our system is nearly the same as the conventional Cas12a system in detecting ssDNA targets, we envision that by integrating with some efficient nucleic acid amplification protocols to produce short RNAs or recycling the *trans*-cleavage products, the facile detection of ultrashort RNAs of ultralow concentrations could be further achieved. Moreover, since the difference between our system and the conventional Cas12a system is merely a splicing of crRNA without additional operational burden, it can be easily combined with many biosensing strategies to promote the development of numerous diagnostic technologies. This innovative approach provides a simple, specific, accurate and promising alternative for detecting ultrashort RNAs, advancing CRISPR/Cas12a-based strategies in the field of molecular diagnostics.

## Supplementary Material

gkaf002_Supplemental_File

## Data Availability

All data supporting the findings of this study are available within the article and its supplementary information or will be made available from the authors upon request.
