# Supplementary material for: ‘Splice-at-will’ Cas12a crRNA engineering enabled direct quantification of ultrashort RNAs
Source: Nucleic Acids Res. 2025 Jan 20;53(2):gkaf002. doi: 10.1093/nar/gkaf002 (PMC11744192; doi:10.1093/nar/gkaf002)
Supplement: gkaf002_Supplemental_File [file gkaf002_supplemental_file.pdf]

"Splice-at-will" Cas12a crRNA engineering enabled direct quantification of ultrashort RNAs

*Xinrui Fei,[1] Chao Lei,[1] Wei Ren,[1] and Chenghui Liu[1,]**

[1]Key Laboratory of Applied Surface and Colloid Chemistry, Ministry of Education; Key Laboratory of Analytical Chemistry for Life Science of Shaanxi Province; School of Chemistry & Chemical Engineering, Shaanxi Normal University, Xi'an 710119, Shaanxi Province, P. R. China.

**Corresponding author. E-mail: liuch@snnu.edu.cn;*

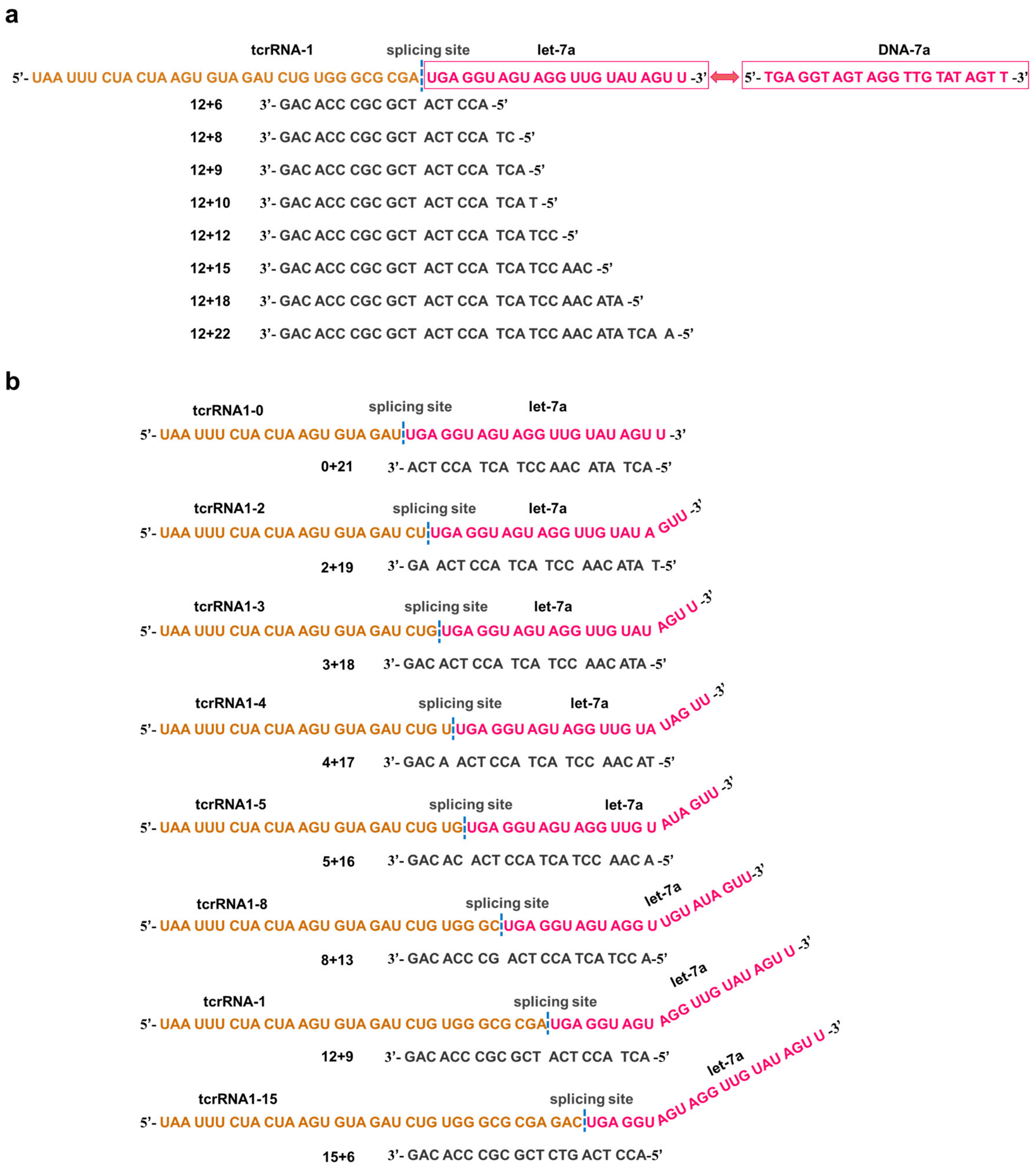

Figure S1. The sequences employed to investigate the effect of combination modes with different splicing lengths on "splice-at-will" crRNA-induced Cas12a activation. The orange part is the tcrRNA sequences, and the pink part is the sequences of let-7a and DNA-7a. The splicing site is represented by a dashed blue line. The black part is the activator sequences. (a) Schematic representation of combining tcrRNA with RNA of different splicing lengths via a series of auxiliary 12+X DNA activators (12nt complementary to tcrRNA and X represents the number of nucleotides complementary to the RNA). (b) Schematic representation of splicing RNA with tcrRNAs of different spacer lengths via a series of X+Y auxiliary DNA activators of 21 nt (X

represents the number of nucleotides complementary to tcrRNA, while Y represents the number of nucleotides complementary to the RNA).

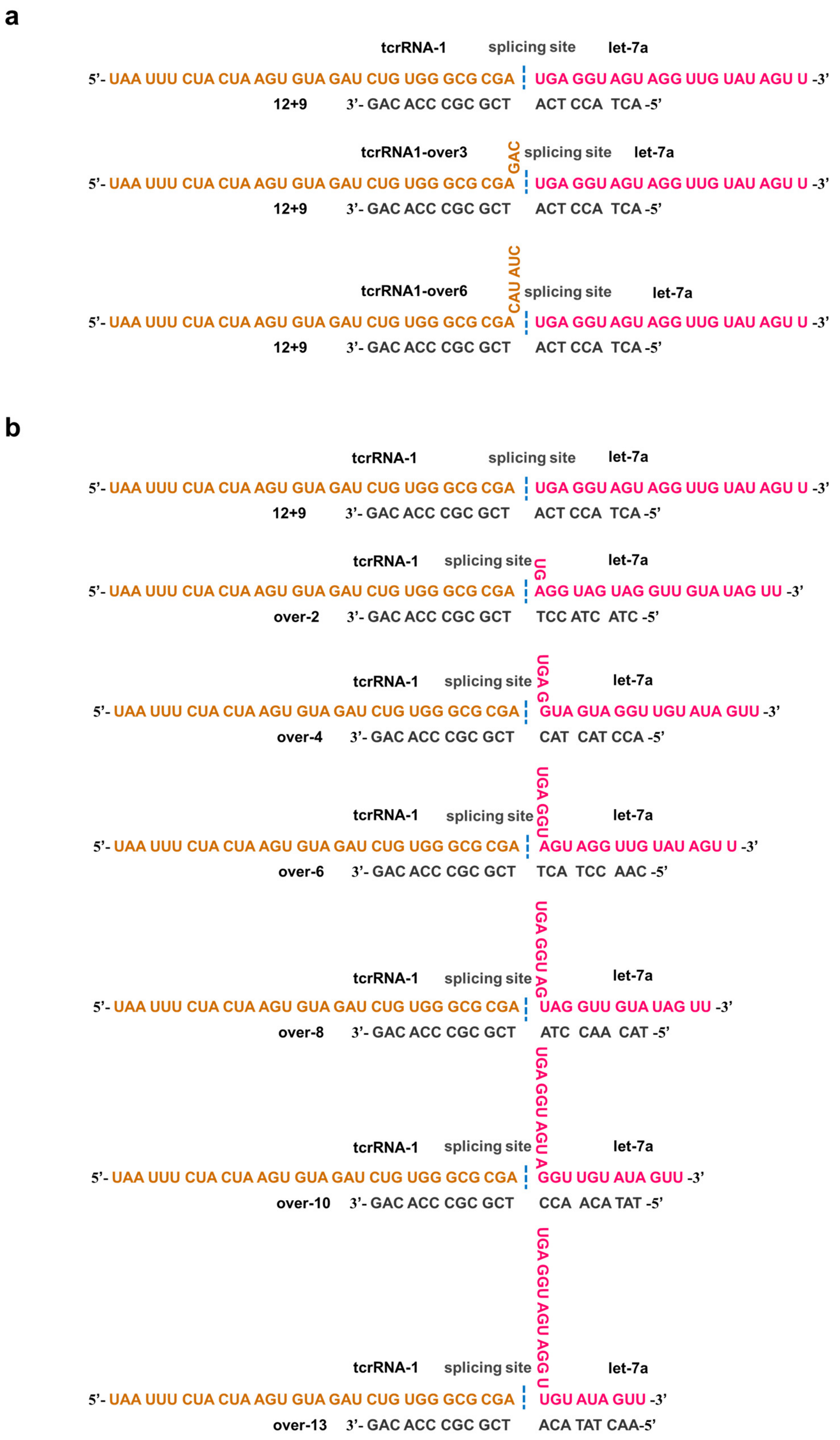
a
tcrRNA-1
splicing site
let-7a
5'- UAA UUU CUA CUA AGU GUA GAU CUG UGG GCG CGA
UGA GGU AGU AGG UUG UAU AGU U -3'
12+9
3'- GAC ACC CGC GCT
ACT CCA TCA -5'
tcrRNA1-over3
GAC
splicing site
let-7a
5'- UAA UUU CUA CUA AGU GUA GAU CUG UGG GCG CGA
UGA GGU AGU AGG UUG UAU AGU U -3'
12+9
3'- GAC ACC CGC GCT
ACT CCA TCA -5'
tcrRNA1-over6
CAU AUC
splicing site
let-7a
5'- UAA UUU CUA CUA AGU GUA GAU CUG UGG GCG CGA
UGA GGU AGU AGG UUG UAU AGU U -3'
12+9
3'- GAC ACC CGC GCT
ACT CCA TCA -5'
b
tcrRNA-1
splicing site
let-7a
5'- UAA UUU CUA CUA AGU GUA GAU CUG UGG GCG CGA
UGA GGU AGU AGG UUG UAU AGU U -3'
12+9
3'- GAC ACC CGC GCT
ACT CCA TCA -5'
tcrRNA-1
splicing site
UG
let-7a
5'- UAA UUU CUA CUA AGU GUA GAU CUG UGG GCG CGA
AGG UAG UAG GUU GUA UAG UU -3'
over-2
3'- GAC ACC CGC GCT
TCC ATC ATC -5'
tcrRNA-1
splicing site
UGA G
let-7a
5'- UAA UUU CUA CUA AGU GUA GAU CUG UGG GCG CGA
GUA GUA GGU UGU AUA GUU -3'
over-4
3'- GAC ACC CGC GCT
CAT CAT CCA -5'
tcrRNA-1
splicing site
UGA GGU
let-7a
5'- UAA UUU CUA CUA AGU GUA GAU CUG UGG GCG CGA
AGU AGG UUG UAU AGU U -3'
over-6
3'- GAC ACC CGC GCT
TCA TCC AAC -5'
tcrRNA-1
splicing site
UGA GGU AG
let-7a
5'- UAA UUU CUA CUA AGU GUA GAU CUG UGG GCG CGA
UAG GUU GUA UAG UU -3'
over-8
3'- GAC ACC CGC GCT
ATC CAA CAT -5'
tcrRNA-1
splicing site
UGA GGU AGU A
let-7a
5'- UAA UUU CUA CUA AGU GUA GAU CUG UGG GCG CGA
GGU UGU AUA GUU -3'
over-10
3'- GAC ACC CGC GCT
CCA ACA TAT -5'
tcrRNA-1
splicing site
UGA GGU AGU AGG U
let-7a
5'- UAA UUU CUA CUA AGU GUA GAU CUG UGG GCG CGA
UGU AUA GUU -3'
over-13
3'- GAC ACC CGC GCT
ACA TAT CAA-5'

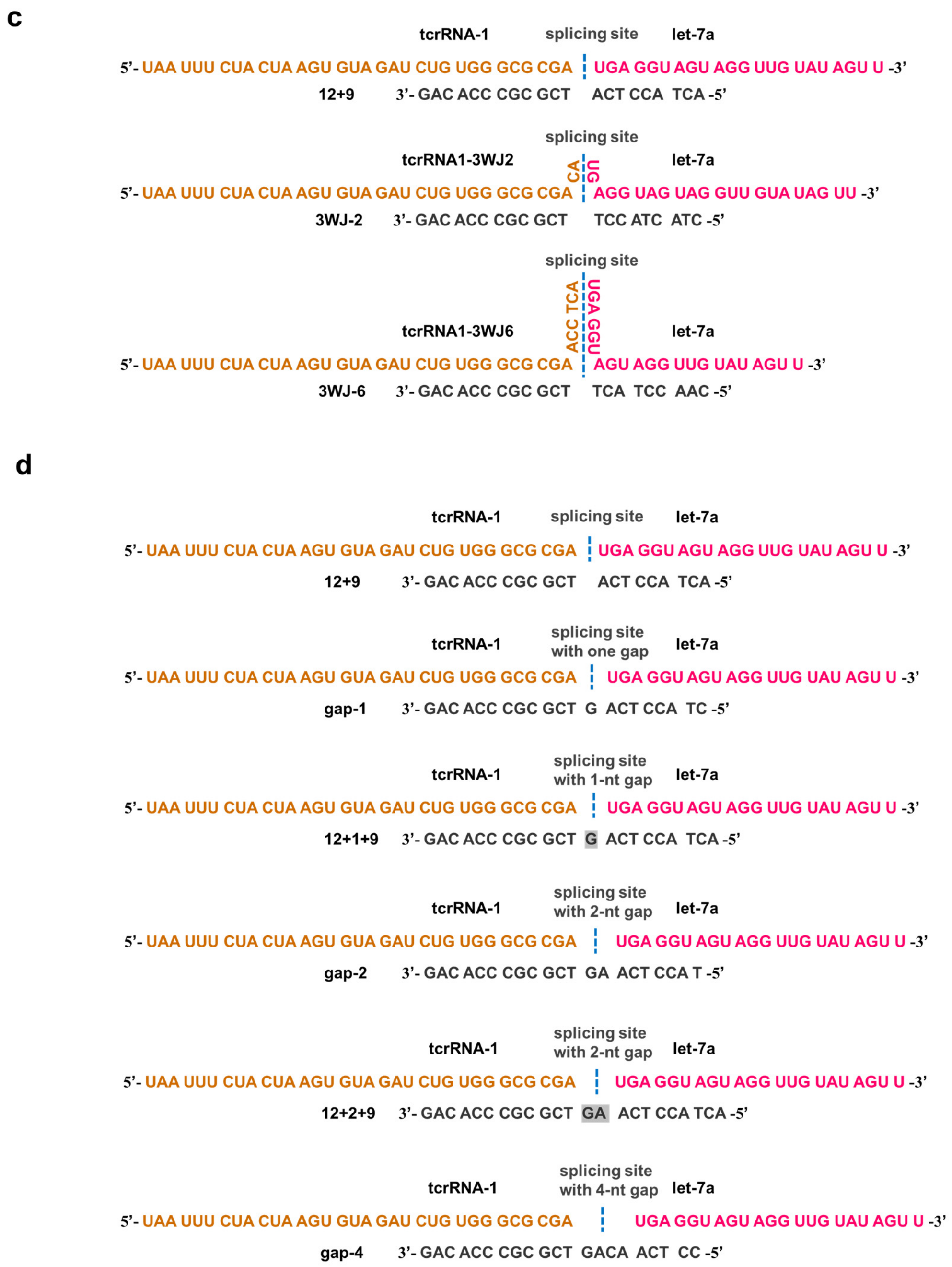

Figure S2. The sequences employed to investigate the effect of structural adjustments at the splice joint site by inserting different lengths of (a) tcrRNA overhang, (b) let-7a overhang, (c) three-way junction, and (d) gap. The orange part is the tcrRNA sequences, and the pink part is the let-7a sequences. The splicing site is represented by a dashed blue line. The black part is the activator sequences. The black part is the activator sequence of 21 nt. The letters in gray shadow are the inserted bases besides 21 nt.

a

tcrRNA-1 splicing site let-7a

5'- UAA UUU CUA CUA AGU GUA GAU CUG UGG GCG CGA ¦ UGA GGU AGU AGG UUG UAU AGU U -3'

12+9 3'- GAC ACC CGC GCT ACT CCA TCA -5'

tcrRNA-2 splicing site let-7a

5'- UAA UUU CUA CUA AGU GUA GAU CUA UGC GCG GGA ¦ UGA GGU AGU AGG UUG UAU AGU U -3'

2-12+9 3'- GAT ACG CGC CCT ACT CCA TCA -5'

tcrRNA-3 splicing site let-7a

5'- UAA UUU CUA CUA AGU GUA GAU CAG AGG GCG GCA ¦ UGA GGU AGU AGG UUG UAU AGU U -3'

3-12+9 3'- GTC TCC CGC CGT ACT CCA TCA -5'

b

tcrRNA-1 splicing site truncated let-7a

5'- UAA UUU CUA CUA AGU GUA GAU CUG UGG GCG CGA ¦ UGA GGU AGU -3'

12+9 3'- GAC ACC CGC GCT ACT CCA TCA -5'

full-length crRNA

5'- UAA UUU CUA CUA AGU GUA GAU CUG UGG GCG CGA UGA GGU AGU -3'

12+9 3'- GAC ACC CGC GCT ACT CCA TCA -5'

Figure S3. The sequences employed to investigate the activation mechanism and efficiency of the "splice-at-will" crRNA. The orange part is the sequences of tcrRNA and full-length intact crRNA, and the pink part is the sequences of let-7a and truncated let-7a. The splicing site is represented by a dashed blue line. The black part is the activator sequence of 21 nt. (a) The sequences used to investigate the universality of the "splice-at-will" crRNA activation effects by changing different tcrRNAs. (b) The sequences used to compare Cas12a relative activity induced by "splice-at-will" crRNA and conventional full-length intact crRNA.

a

tcrRNA-1 splicing site let-7a

5’- UAA UUU CUA CUA AGU GUA GAU CUG UGG GCG CGA ¦ UGA GGU AGU AGG UUG UAU AGU U -3’

12+9 3’- GAC ACC CGC GCT ACT CCA TCA -5’

b

UGA GGU AGU AGGU

tcrRNA-1 splicing site let-7a

5’- UAA UUU CUA CUA AGU GUA GAU CUG UGG GCG CGA ¦ UGU AUA GUU -3’

over-13 3’- GAC ACC CGC GCT ACT CCA TCA -5’

let-7a 5’- UGAGGUAGUAGGU UGU AUA GUU -3’

let-7b 5’- UGA GGU AGU AGGU UGU GUG GUU -3’

let-7c 5’- UGAGGUAGUAGGU UGU AUG GUU -3’

let-7d 5’- AGAGGUAGUAGGU UGC AUA GU -3’

miRNA-21 5’- UAGCUUAUCAGAC UGA UGU UGA -3’

miRNA-125b 5’- UCCCUGAGACCCU AAC UUG UGA -3’

c

tcrRNA-1 splicing site miRNA-17

5’- UAA UUU CUA CUA AGU GUA GAU CUG UGG GCG CGA ¦ CAA AGU GCU UAC AGU GCA GGU AG -3’

17-12+9 3’- GAC ACC CGC GCT GTT TCA CGA -5’

tcrRNA-1 splicing site P8 6s-1

5’- UAA UUU CUA CUA AGU GUA GAU CUG UGG GCG CGA ¦ GUU CGG UC -3’

p8-12+9 3’- GAC ACC CGC GCT CAA GCC AG -5’

Figure S4. The sequences employed to detect miRNA and ultrashort RNA by the “splice-at-will” system and distinguish single base differences therein. The orange part is the tcrRNA sequences, and the blue part is the sequences of RNA targets (let-7a, miRNA-17, and p8 6s-1) and other miRNAs (let-7b, let-7c, let-7d, miRNA-21 and miRNA-125b). The black part is the activator sequences. The yellow highlighted letters are the mismatched bases. (a) The sequence used in let-7a detection. (b) The sequence used in the single base distinguishing of let-7a families. (c) The sequence used in miRNA-17 and p8 6s-1 detection.

a

Cas13a crRNA-7a

5'- GGG AUU UAG ACU ACC CCA AAA ACG AAG GGG ACU AAA AC AAC UAU ACA ACC UAC UAC CUC A -3'

let-7a 3'- UUG AUA UGU UGG AUG AUG GAG U -5'

Cas13a crRNA-p8

5'- GGG AUU UAG ACU ACC CCA AAA ACG AAG GGG ACU AAA AC GAC CGA AC -3'

p8 6s-1 3'- CUG GCU UG-5'

b

Cas12a crRNA-7a

5'- UAA UUU CUA CUA AGU GUA GAU AAC UAU ACA ACC UAC UAC CUC A -3'

let-7a 3'- UUG AUA UGU UGG AUG AUG GAG U -5'

Cas12a crRNA-p8

5'- UAA UUU CUA CUA AGU GUA GAU GAC CGA AC -3'

p8 6s-1 3'- CUG GCU UG-5'

Figure S5. The sequences employed to detect miRNA and ultrashort RNA by conventional CRISPR/Cas systems. The orange part is the sequences of crRNA, and the pink part is the sequences of RNA targets (let-7a and p8 6s-1). (a) The sequences used in let-7a and p8 6s-1 detection by conventional Cas13a system. (b) The sequences used in let-7a and p8 6s-1 detection by conventional Cas12a system.

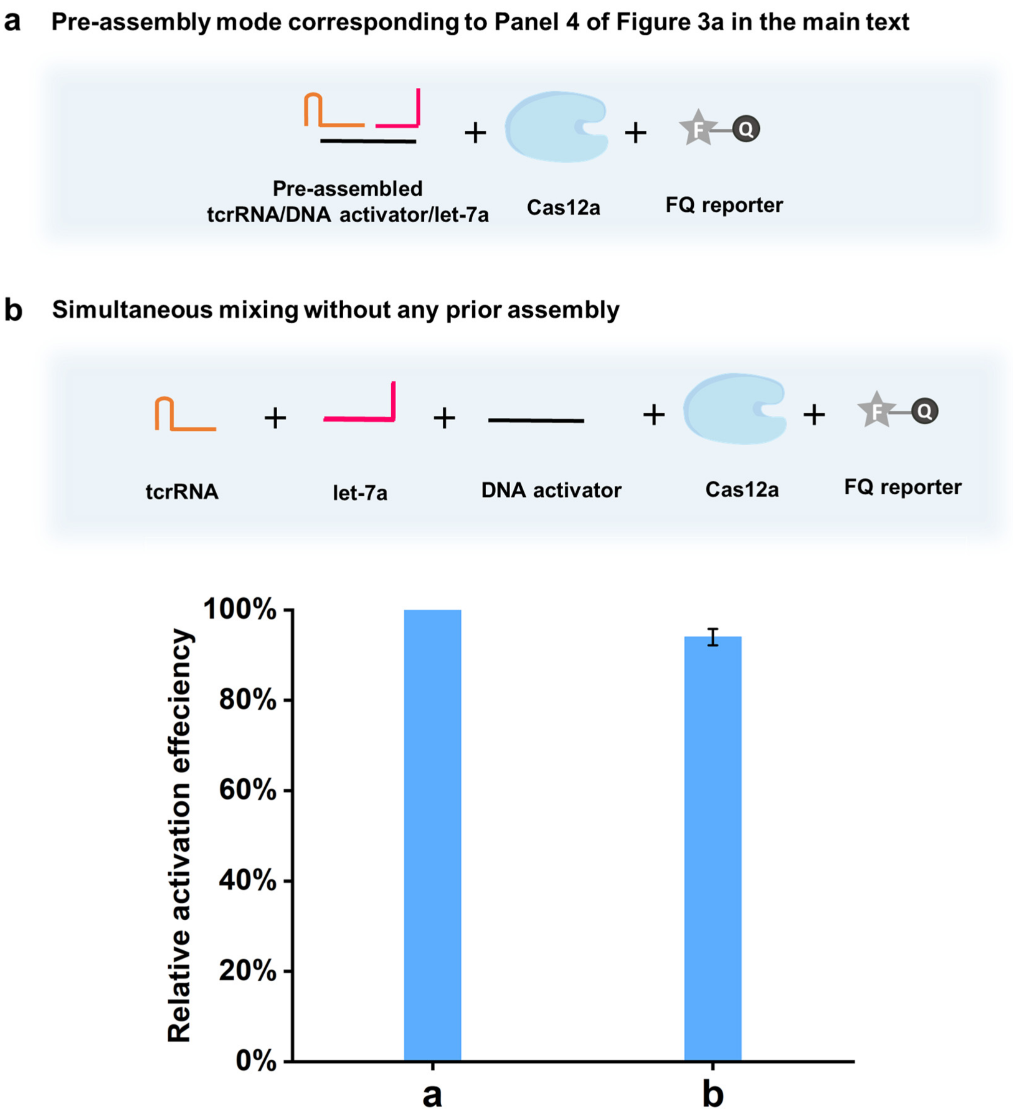

Figure S6. The relative activation efficiency of simultaneous mixing to pre-assembly mode of the "splice-at-will" system. (a) The schematic diagram of pre-assembly mode corresponding to panel 4 of Figure 3a in the main text. (b) The schematic diagram of simultaneous mixing without any prior assembly. Error bars were calculated from triplicate experiments.

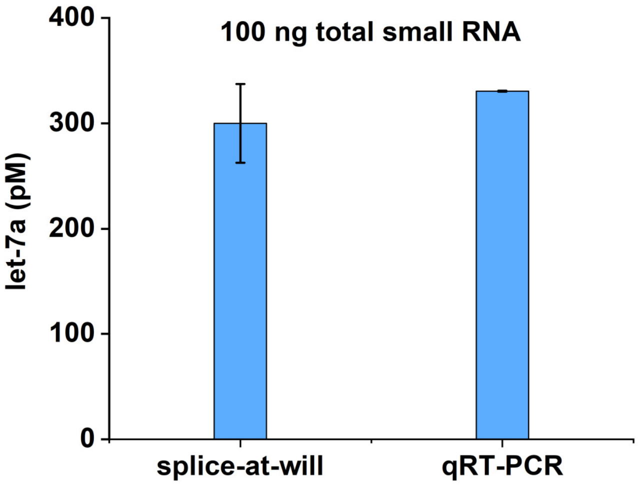

Figure S7. Detection of let-7a in total small RNA sample by splice-at-will sensing system and RT-PCR. (RT-PCR protocol: the 10 μL whole reaction system includes the following two steps: In the reverse transcription reaction, target miRNA (or total small RNA sample), 1 × RT buffer (50 mM Tris-HCl, 75 mM KCl, 3 mM $MgCl_2$), 250 μM dNTPs, 4 U/μL ProtoScrip II reverse transcriptase, 50 nM RT-Probe (5’-GTCGTATCCAGTGCAGGGTCCGAGGTATTCGCACTGGATACGACAACTA-3’) and 0.4 U/μL RNase inhibitor was treated with following conditions: 30 min at 16 °C, 30 min at 42 °C, 5 min at 85 °C and then held at 4 °C. In the Quantitative real-time PCR analysis steps, the above transcription product, 200 nM forward primer (5’-GCCGCTGAGGTAGTAGGTTGTA-3’), 200 nM reverse primer (5’-GTGCAGGGTCCGAGGT-3’), 250 μM dNTPs, 0.4 × SYBR Green I, 0.5 U JumpStartTM Taq DNA Polymerase, and 1 × PCR buffer (10 mM Tris-HCl, 50 mM KCl, 1.5 mM $MgCl_2$, 0.001(w/v) gelatin, pH 8.3) was incubated in a StepOne Real-Time PCR System (Applied Biosystems, USA) according to the following thermal cycling conditions: hot start at 95 °C for 2 min, followed by 50 cycles of 95 °C for 15 s, and 60 °C for 1 min).

Table S1. Customized nucleic acid sequences used in this study.

Name	Sequences (5'-3')
tcrRNA-1	UAAUUUCUACUAAGUGUAGAUCUGUGGGCGCGA
let-7a	UGAGGUAGUAGGUUGUAUAGUU
DNA-7a	TGAGGTAGTAGGTTGTATAGTT
12+6	ACTACCTCATCGCGCCCACAG
12+8	CTACCTCATCGCGCCCACAG
12+9	ACTACCTCATCGCGCCCACAG
12+10	TACTACCTCATCGCGCCCACAG
12+12	CCTACTACCTCATCGCGCCCACAG
12+15	CAACCTACTACCTCATCGCGCCCACAG
12+18	ATACAACCTACTACCTCATCGCGCCCACAG
12+22	AACTATACAACCTACTACCTCATCGCGCCCACAG
tcrRNA1-0	UAAUUUCUACUAAGUGUAGAU
0+21	ACTATACAACCTACTACCTCA
tcrRNA1-2	UAAUUUCUACUAAGUGUAGAUCU
2+19	TATACAACCTACTACCTCAAG
tcrRNA1-3	UAAUUUCUACUAAGUGUAGAUCUG
3+18	ATACAACCTACTACCTCACAG
tcrRNA1-4	UAAUUUCUACUAAGUGUAGAUCUGU
4+17	TACAACCTACTACCTCAACAG
tcrRNA1-5	UAAUUUCUACUAAGUGUAGAUCUGUG
5+16	ACAACCTACTACCTCACACAG
tcrRNA1-8	UAAUUUCUACUAAGUGUAGAUCUGUGGGC
8+13	ACCTACTACCTCAGCCCACAG
tcrRNA1-15	UAAUUUCUACUAAGUGUAGAUCUGUGGGCGCGAGAC
15+6	ACCTCAGTCTCGCGCCCACAG
tcrRNA1-over3	UAAUUUCUACUAAGUGUAGAUCUGUGGGCGCGAGAC
tcrRNA1-over6	UAAUUUCUACUAAGUGUAGAUCUGUGGGCGCGAGAUAUC
over-2	CTACTACCTTCGCGCCCACAG
over-4	ACCTACTACTCGCGCCCACAG
over-6	CAACCTACTTCGCGCCCACAG
over-8	TACAACCTATCGCGCCCACAG
over-10	TATACAACCTCGCGCCCACAG
over-13	AACTATACATCGCGCCCACAG
tcrRNA1-3WJ2	UAAUUUCUACUAAGUGUAGAUCUGUGGGCGCGACA

3WJ-2	CTACTACCTTCGCGCCCACAG
tcrRNA1-3WJ6	UAAUUUCUACUAAGUGUAGAUCUGUGGGCGCGAACCTCA
3WJ-6	CAACCTACTTCGCGCCCACAG
gap-1	CTACCTCAGTCGCGCCCACAG
12+1+9	ACTACCTCAGTCGCGCCCACAG
gap-2	TACCTCAAGTCGCGCCCACAG
12+2+9	ACTACCTCAAGTCGCGCCCACAG
gap-4	CCTCAACAGTCGCGCCCACAG
tcrRNA-2	UAAUUUCUACUAAGUGUAGAUCUAUGCGCGGGA
2-12+9	ACTACCTCATCCCGCGCATAG
tcrRNA-3	UAAUUUCUACUAAGUGUAGAUCAGAGGGCGGCA
3-12+9	ACTACCTCATGCCGCCCTCTG
truncated let-7a	UGAGGUAGU
full-length crRNA	UAAUUUCUACUAAGUGUAGAUCUGUGGGCGCGAUGAGGUAGU
let-7b	UGAGGUAGUAGGUUGUGUGGUU
let-7c	UGAGGUAGUAGGUUGUAUGGUU
let-7d	AGAGGUAGUAGGUUGCAUAGU
miRNA-21	UAGCUUAUCAGACUGAUGUUGA
miRNA-125b	UCCCUGAGACCCUAACUUGUGA
miRNA-17	CAAAGUGCUUACAGUGCAGGUAG
17-12+9	AGCACTTTGTCGCGCCCACAG
p8 6s-1	GUUCGGUC
P8-12+9	GACCGAACTCGCGCCCACAG
Cas12a crRNA-7a	UAAUUUCUACUAAGUGUAGAUAACUAUACAACC UACUACCUCA
Cas12a crRNA-p8	UAAUUUCUACUAAGUGUAGAUGACCGAAC
Cas13a crRNA-7a	GGGAUUUAGACUACCCCAAAAACGAAGGGGACUAAAACAACUAUA CAACC UACUACCUCA
Cas13a crRNA-p8	GGGAUUUAGACUACCCCAAAAACGAAGGGGACUAAAACGACCGAA C

Table S2. Recovery experiments in total small RNA sample.

Sample	Amount of p8 6s-1	Average value	Recovery
20 ng total small RNA (3 parallel determination)	undetectable (samples 1, 2, and 3)	-	-
20 ng total small RNA +100 pM p8 6s-1 (3 parallel determination)	96.0 pM (spiked sample 1)<br>94.4 pM (spiked sample 2)<br>98.2 pM (spiked sample 3)	96.2 pM	96.2%
